# Computed Tomography Image Features under Denoising Algorithm for Benign and Malignant Diagnosis of Renal Parenchymal Tumor

**DOI:** 10.1155/2022/5871385

**Published:** 2022-05-27

**Authors:** Zhongxiao Zhang, Zehua Wang

**Affiliations:** ^1^Department of Urology, Qilu Hospital (Qingdao), Shandong University, Qingdao 266035, Shandong, China; ^2^Department of Urology, Qilu Hospital, Shandong University, Jinan 250012, Shandong, China

## Abstract

To improve the quality of computed tomography (CT) images and provide help for benign and malignant diagnosis of renal parenchymal tumors, the independent component analysis (ICA) denoising algorithm was used. An improved ICA X-ray CT (X-CT) medical image denoising algorithm was proposed. ICA provided a higher signal-to-noise ratio for CT image denoising. Forty patients with renal tumor were selected as the observation group. The CT image performance of patients was evaluated by the denoising algorithm and compared with the wavelet transform algorithm, and the peak signal-to-noise ratio of the proposed algorithm was analyzed and compared. The results showed that among the 40 patients with renal tumors, 12 were renal clear cell carcinoma cases and 28 were cystic renal carcinoma cases. The accuracy of the enhanced CT image was 93.8%, and that of the CT image using the denoising algorithm was 96.3%; the difference between the two was significant (*P* < 0.05). The peak signal-to-noise ratio (PSNR) of the algorithm proposed was higher than the PSNR values of CT and noisy images. The PSNR of the proposed algorithm was significantly higher than that of mean filtering. The root mean square error (RMSE) algorithm of the proposed algorithm was significantly lower than that of the mean algorithm in image data processing (*P* < 0.05), which showed the superiority of the proposed algorithm. Enhanced CT can be staged significantly. In conclusion, the algorithm had a significant effect on the edge contour of detailed features, and the accuracy of CT images based on intelligent calculation was significantly higher than that of conventional CT images for benign and malignant renal parenchyma tumors, which was worth promoting in clinical diagnosis.

## 1. Introduction

Renal malignant tumor accounts for about 3% of all malignant tumors, which is a kind of primary renal cells and a relatively rare malignant tumor. Malignant tumors often have necrosis, ulceration, bleeding, and other conditions. However, benign tumors are mostly characterized by secondary changes, such as hemorrhage, necrosis, and ulcers [1, 2]. Malignant tumors can cause organ failure, infection, invasion, and destruction of tissues and organs [3–5]. Renal solid tumors are more likely to develop into renal cancer, and magnetic resonance imaging (MRI), enhanced computed tomography (CT), and renal puncture biopsy can be selected for detection without definite examination. For benign tumors, resection can be considered; for malignant tumors, radical resection is required [6]. As the disease progresses, the patient may develop hematuria, pain, or a mass. Any of these symptoms may indicate that the tumor has advanced. In adults, hematuria is an early and common symptom. Hematuria is mostly visible, whereas some hematuria can only be seen under a microscope. It is generally painless in patients when hematuria occurs. Hematuria is mostly intermittent and can often stop by itself [7]. The commonly used imaging methods for diagnosis of renal tumor include B ultrasound, X-ray, CT, and MRI. The accuracy of CT/MRI was the highest in a single examination, followed by B ultrasound. The accuracy of CT for renal cancer staging was better than that of X-ray and B ultrasound. CT is a noninvasive imaging method that can distinguish vascular, inflammatory, and cystic lesions.

Compared with traditional X-ray examination, CT has a higher density resolution and can clearly display soft tissues, joints, and other parts. With the continuous updation of high-tech medical equipment, the development of CT images is also changing rapidly. Studies on intelligent algorithms combined with CT images have mushroomed, which has expanded the scope of examination and improved the diagnostic level [8, 9]. However, interference of external signals often occurs in medical images, and the signals will be weakened during transmission. The image transmission process will be affected by imaging equipment, resulting in insufficient image clarity. Moreover, the feature display is not obvious, and the focus is not prominent, which cannot adapt well to the automatic analysis of the machine. The image is noisy and ambiguous, which makes it impossible to accurately locate the relevant lesions, thus reducing the judgment rate of doctors [10, 11]. X-ray CT medical imaging can filter out the noise in the image and provide a good visual environment for the analysis/observation of the image. X-CT images can eliminate irrelevant information in the image, enhance the detectability of relevant information by filtering out noise, and maintain clear contour lines while removing noise. The improvement of image scanning technology is helpful to reduce the influence of artifact and noise on CT examination, reduce X-ray radiation, and improve the quality of CT image. After the image is processed by the intelligent algorithm, it is conducive to data extraction, highlighting target features, and providing more reliable information [12–14].

Traditional denoising algorithms mainly include median filter, mean filter, and spatial wiener. In the process of noise elimination, these traditional denoising algorithms cannot retain the details of the image well and the image edge information is damaged. Thus, they cannot achieve a good denoising effect, which directly affects the diagnosis of the disease by doctors [15, 16]. The image denoising method of wavelet transform can save the details of the image well and retain most of the signal information, but there will be a fuzzy situation at the edge of the image. The X-CT image algorithm can recover useful real information, eliminate irrelevant information, and enhance the detectability of relevant information after noise filtering [17, 18]. Independent component analysis (ICA) is an efficient blind separation method, which can treat the polluted image as a mixture of the source image and noise during image denoising. Regardless of the noise intensity, it can be filtered out by some analysis methods and the restored image can retain the loss of image details. At present, ICA has been applied in face and character recognition [19], noise filtering [20], feature extraction [21], and other aspects, showing good results. The basic theoretical framework of ICA has been perfected, but there are still many problems that need to be further discussed, such as the ICA model with noise. The assumption that the model has many uncertainties needs to be optimized.

Therefore, the ICA denoising algorithm was used to denoise CT images, and separated original images and noise images were obtained by ICA to obtain a higher peak signal-to-noise ratio. The denoised images were used to accurately diagnose the benign and malignant renal substantial tumors, providing a reference for clinical diagnosis and treatment of diseases.

## 2. Data and Methods

### 2.1. Clinical Data

Forty patients with renal tumor admitted to hospital were included in the observation group, including 27 males and 13 females, aged 26–69 years. All patients underwent CT examination before surgery to accurately understand the size of tumors. Another forty healthy patients during the same period of physical examination were selected as the control group, including 21 males and 19 females, aged 27–70 years. The clinical data of the two groups of patients were complete. There was no significant difference in general data between the two groups (*P* > 0.05), indicating comparability. This study had been approved by the ethics committee of the hospital, and all the patients who participated signed informed consent.

Inclusion criteria were as follows: (i) patients with complete clinical data; (ii) patients with skin lesions, (iii) patients with no immune system diseases and no infectious diseases; (iv) patients who volunteered to join this research. Exclusion criteria were are follows: (i) patients with incomplete clinical data; (ii) patients unwilling to participate in this study; (iii) patients with heart, liver, kidney, and hematopoietic system diseases, diabetes, and other diseases; (iv) patients with congenital lesions; (v) patients with sites that did not meet the inclusion criteria.

### 2.2. Enhanced CT Scan

Patients were scanned with a spiral CT machine. The contrast agent was injected into the cubital vein with a high-pressure syringe at a rate of 3 mL/s in a single stage, with an injection volume of 90–100 mL. CT scans were performed from the patient's liver to the upper ureter. The first stage of the whole liver scan was performed 25–30 seconds after the contrast agent injection, the second stage was performed 60–70 seconds later, and the third stage was performed 120–180 seconds later. The pattern of lesion enhancement was recorded during the scan, and all the data were imported into a computer. The examination of the enhanced CT was performed by two physicians, and the data of departure were analyzed. The physician should have at least five years of experience and be able to master the procedure. If there were different opinions during the diagnosis process, the final judgment should be made after discussion.

### 2.3. Wavelet Transform Denoising Algorithm

**Figure 1:**
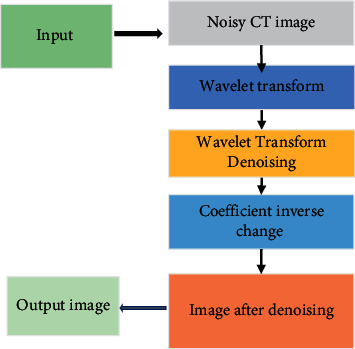
Wavelet transform denoising flowchart.

The wavelet transform denoising method processes the image containing noise through the corresponding regular wavelet coefficients. According to different coefficient characteristics of the processing, it can retain the wavelet coefficient of the image signal. The wavelet theory image denoising process mainly includes the wavelet transform, the wavelet coefficient processing, and finally the inverse transformation of the coefficient after processing (Figure 1).

X-CT uses the X-ray beam received by the detector, and the X-ray signal is converted into an electrical signal, the photoelectric capacity is then converted into an electrical signal, digital analog is converted into a digital signal, and the digital signal is processed by a computer. X-CT medical images are arranged in a matrix, and the pixels reflect the X-ray absorption coefficient. The image sizes range from 0.5 × 0.5 mm to 1.0 × 1.0 mm. The X-CT devices corresponding to different pixel sizes are different, and the number and size of the images are also different. For images with high resolution, the number of pixels is more and the information stored is richer. The high resolution of X-ray CT images is also a significant feature, which can clearly present the pathological images under a good anatomical image background. X-CT image quality is also affected by a variety of factors; the main factors include scanning technical parameters, mechanical calibration, and image quality parameters, and these factors also restrict each other. Noise is an unpredictable random signal produced by human visual organs. To analyze image noise from the point of view of mathematics, a mathematical model needs to be established and the function information expressed by the function is used to degrade the noise image. If the signal under the influence of noise is *z* (*x, y*), *n* (*x, y*) represents noise, and the output signal is represented by *g* (*x, y*), then the mean value of the total intensity of the image noise can be expressed as follows:

(1) $$\begin{array}{c} \mu =E\left(\text{n}\left(x,y\right)\right)+\frac{1}{\text{A}\times \text{B}}\sum_{x=1}^{A}\sum_{y-1}^{B}\text{n}\left(x,\text{y}\right). \end{array}$$

*A* is the row of the image matrix, and *B* is the column of the image matrix.

The variance of image noise is expressed by the following equation, that is, the fluctuation of image noise intensity:

(2) $$\begin{array}{c} \eta^{2}=E{\left(\text{n}\left(x,y\right)-\mu \right)}^{2}+\frac{1}{\text{A}\times \text{B}}\sum_{x=1}^{A}\sum_{y-1}^{B}{\left[\text{n}\left(x,\text{y}\right)-\mu \right]}^{2}. \end{array}$$

Two mathematical models represent image degradation, (3) represents additive noise, and (4) represents multiplicative noise. In the actual image, the noise and the image signal are independent of each other. The calculation of multiplicative noise is to transform the logarithmic change into additive noise, and the calculation process is relatively easy. After the image is denoised, the index can be selected and then converted.

(3) $$\begin{array}{c} \text{g}\left(x,y\right)=z\left(x,y\right)+n\left(x,y\right), \end{array}$$

(4) $$\begin{array}{c} \text{g}\left(x,y\right)=z\left(x,y\right)\left(1+n\left(x,y\right)\right). \end{array}$$

Quantum noise and photon noise are the main noises in X-CT images, and both can use Gaussian white noise as the model. The one-dimensional probability density function of Gaussian noise can be expressed as follows:

(5) $$\begin{array}{c} P\left(Q\right)={\frac{1}{\sqrt{2\pi }\varphi }}^{\text{e}-{\left(\text{q}-\mu \right)}^{2}/2\varphi^{2}}. \end{array}$$

Here, *Q* represents the gray level *φ* of noise points and the standard deviation of *Q*, *φ*^2^ represents the variance of *Q*, and *μ* represents the mean value of *Q*. The noise of different pixels in an image is irrelevant.

**Figure 2:**
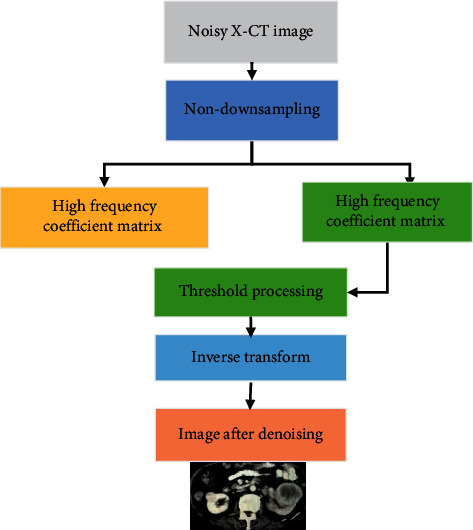
Flow chart of the X-CT denoising algorithm.

The algorithm flow chart is shown in Figure 2. The image containing noise is first sampled and then divided into a low-frequency coefficient matrix and a high-frequency coefficient matrix, and the inverse transformation is processed by the threshold value. Finally, the denoised image is obtained.

### 2.4. ICA Image Denoising Algorithm

The objective function equation of the ICA image algorithm is expressed as follows, and the calculation is easy to achieve universally:

(6) $$\begin{array}{c} U\left(y\right)={\left|E_{\text{y}}\left[G\left(y\right)\right]-E_{\text{y}}\left[G\left(v\right)\right]\right|}^{p}. \end{array}$$

*V* is a standard Gaussian random variable, the index *p* = log2, and *G* represents a nonquadratic and sufficiently smooth function. The nonquadratic function *G* is expressed as follows:

(7) $$\begin{array}{c} G_{1}\left(\mu \right)=\log\cosh a_{1}\mu . \end{array}$$

Or, *G* is expressed as follows:

(8) $$\begin{array}{c} G_{1}\left(\mu \right)=\exp\left(-\frac{{\text{a}}_{2}\mu^{2}}{2}\right). \end{array}$$

For the Sub-Gaussian variable, take *G*1. For Sub-Gaussian, take *G*2.

The iterative process of image preprocessing is expressed as follows:

(9) $$\begin{array}{c} W\left(\text{k}\right)=E\left\{xg\left[w{\left(k-1\right)}^{T}x\right]\right\}-E\left\{g\left[w{\left(k-1\right)}^{T}x\right]\right\}w\left(k-1\right). \end{array}$$

After each iteration, a new W is obtained for normalization and the equation can be expressed as follows:

(10) $$\begin{array}{c} w\left(k\right)=-\frac{w\left(k\right)}{\left\|w\left(k\right)\right\|}. \end{array}$$

### 2.5. Evaluation of Image Denoising Performance

X-CT medical imaging requires high image quality, and the loss of a little detail will have a certain impact on diagnosis. Enhancing the image information of interest is an important part of image processing, and qualitative and quantitative analysis and evaluation are carried out after image processing. Generally, subjective image analysis is based on people's sense and vision, with different reference standards and some differences in image cognition (Table 1).

Quantitative indexes were selected for objective evaluation. The root mean square error (RMSE), peak signal-to-noise ratio (PSNR), and mean square error (MSE) are some commonly used objective evaluation indexes, which can reflect the gray difference between the original image and the processed image. PSNR provides information and noise ratio for the image, while RMSE is the gray difference between the denoised image and the original image. The greater the signal-to-noise ratio of the image, the better the image quality.

(11) PSNR=lolL21/AB∑i=1A∑j=1Bzij−zij'RMSE=1AB∑i=1A∑j=1Bzij−zij'2MSE=1AB∑i=1A∑j=1Bzij−zij'2.

Here, *L* is the gray value range, *Z*_*ij*_ is the value of the original image at (*i, j*), *A* is the number of pixels in the image in the *y* direction, and *B* is the number of pixels in the image in the *x* direction.

The experiment was carried out in the Windows operating system, the language was Mlab7.13 compilation environment, with 8 GB internal storage, the main frequency was 3.0 GHz (Inter quad-core), and the image size was 512 × 521.

### 2.6. Statistical Methods

SPSS 22.0 was used for data processing, and analysis methods were selected according to different situations. The paired sample *t* test was used to compare the changes of patient-related indicators, the independent sample *t* test was used for intergroup differences, ANOVA was used for multiple data, and *P* < 0.05 was used to determine whether there was statistical significance.

## 3. Results

### 3.1. Clinical Data Statistics of Patients

After CT examination, the malignant tumor showed uneven linear enhancement and increased pseudocapsule display, with liquefaction necrosis. After comparison, there was no significant difference in the basic information between the two groups (*P* > 0.05, Table 2).

### 3.2. Image Denoising Comparison with Different Methods

**Figure 3:**
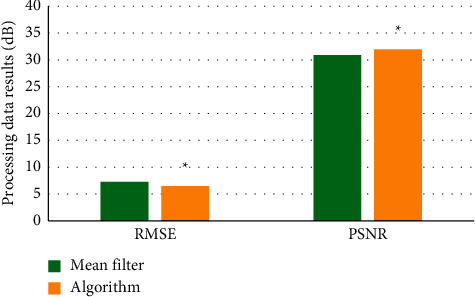
Comparison of image denoising. ^*∗*^A significant difference, *P* < 0.05.

**Figure 4:**
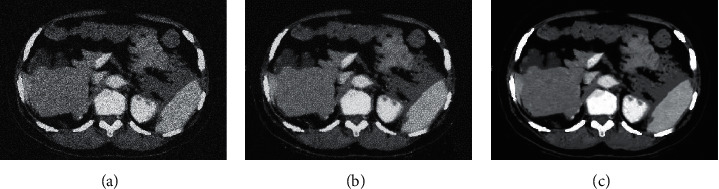
Comparison of CT images. (a) The image before CT enhancement. (b) The CT image after PSNR denoising. (c) The CT image after mean filtering algorithm denoising.

The result of mean filtering algorithm was compared with the image filtering results of the algorithm used in this work, and the results are shown in Figure 3. The PSNR of the proposed algorithm was significantly higher than that of the mean filtering algorithm, and the RMSE of the proposed algorithm was significantly lower than that of the mean filtering algorithm for image data processing (*P* < 0.05), indicating the superiority of the proposed algorithm.  Figure 4(a) shows the original image with obvious fine particles. Compared with the original image, Figures 4(b) and 4(c) had a lighter sense of granularity and significantly enhanced clarity.

### 3.3. PSNR Comparison of Denoising Algorithms

**Figure 5:**
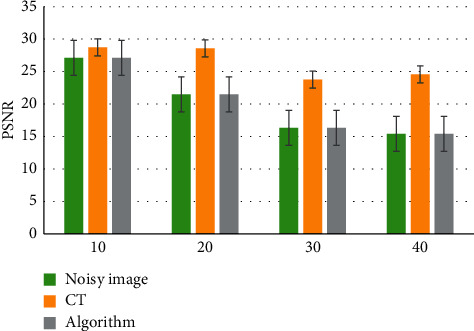
PSNR comparison of different algorithms.

Different sigma values (10, 20, 30, and 40) were selected to compare the PSNR of the algorithm, and the results are shown in Figure 5. The value of the proposed algorithm was higher than the PSNR values of CT and noisy images, which also showed that the algorithm had a significant effect on the detailed feature edge contour.

### 3.4. Accuracy

The CT images of enhanced CT and those that underwent denoising algorithm treatment were compared, and the diagnostic results were analyzed as shown in Table 3. The accuracy of the enhanced CT was 93.8%, while that of CT images using the denoising algorithm was 96.3%. The difference between the two was significant (*P* < 0.05).

### 3.5. Contrast-Enhanced CT Images

**Figure 6:**
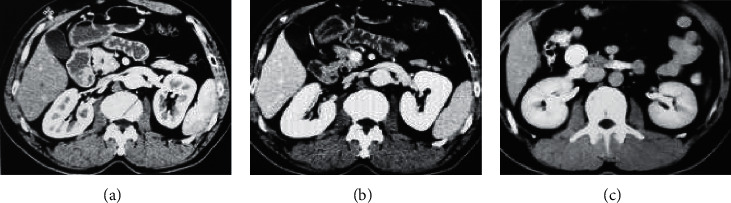
Enhanced CT images.

The CT value was 20 Hu higher than that of the plain scan. The contrast agent in the medulla phase was partially excluded. The pseudocapsule sign, in which the renal tissue around the tumor was deformed and fibrotic under pressure, shows thin linear shadows without enhancement. Persistent filling defects of the renal vein and inferior vena cava were tumor emboli.  Figure 6(a) shows the dermal medulla junction (30 s), Figure 6(b) shows the dermal medulla enhancement (100 s), and Figure 6(c) shows the development period of the collection system (5 min).

## 4. Discussion

Imaging examination is the most basic method for disease detection. With the continuous progress of the times, examination technology is also developing. The emergence of enhanced CT has further improved the accuracy of ultrasound diagnosis, which plays a very important role in the diagnosis of malignant tumor-related diseases [22]. The earlier a tumor disease is discovered, the easier it is to be treated. More attention is paid to renal malignant tumors, and the accuracy, specificity, and sensitivity of tumor detection are also improving [23]. CT renal scan is usually performed by plain CT scan followed by enhanced CT scan. There are two stages in the enhanced scan: the renal cortex medulla stage (arterial stage) and the renal parenchyma stage (venous stage). In the medulla stage, because of the low density of the medulla, the boundary between medulla and cortex is very clear. In the renal parenchymal stage, the medulla images tend to be uniform due to the gradual influx of contrast agents into the cortex. Kang et al. [24] used CT to differentiate renal clear cell sarcoma from nephroblastoma, and the specificity was 86%, which was of significant value in differentiating renal clear cell sarcoma. In this study, the optimized algorithm based on denoising can distinguish irregular and difficult inflammatory responses with clearer two-dimensional boundaries. The classification of cystic renal carcinoma was proposed by radiologist Hartman et al. [25] in 1986, which mainly refers to renal cell carcinoma with cystic changes in imaging and gross pathology, as well as renal cell carcinoma with cystic changes found during surgery. According to pathophysiology, there are four types of cystic renal cell carcinoma, namely, cystic renal cell carcinoma, unilocular cystic renal cell carcinoma, cystic changes in renal cell carcinoma, and simple cyst canceration. The tumor is a cystic growth, derived from a cyst or a canceration of the cyst, and is absorbed by hemorrhage to form a cyst. In this study, the accuracy of enhanced CT was 93.8%, and the accuracy of the denoising algorithm was 96.3%. The accuracy of each algorithm did not reach 100%. In clinical practice, CT imaging still needs to be considered from many aspects and multiple case treatment plans should be referred to. The intelligent CT imaging algorithm can diagnose benign and malignant tumors. In case of poor conventional ultrasound diagnosis or suspected cases, the intelligent algorithm should be used to present auxiliary diagnosis.

There will be different degrees of reinforcement in the essence stage, which is expressed as “fast forward slow retreat” or “fast forward fast retreat.” The enhancement of the tumor smooth muscle and vascular components was obvious, showing onion skin, grid, and vortex enhancement. There were 12 patients with renal clear cell carcinoma which originated from the renal parenchyma, easily invaded adjacent tissue, often metastasized, could be calcified, and had obvious heterogeneous enhancement. Cortical enhancement in some renal cell carcinoma may approach or be higher than the renal cortex. Cystic nephroma showed complete intracapsular segmentation, no obvious mural nodules, equal or slightly low density, and mild to moderate continuous enhancement. The cystic contents were mainly myxomatous and closely connected with fibrous septa. Cystic renal carcinoma changes occurred in 5–7% of renal carcinomas and in 0.5% of renal cysts associated with renal carcinoma. In the first stage, the tumor was confined to the renal capsule with prominent limitations but smooth margins. In the second stage, the tumor was prominent, with a rough surface and a protrusion of 1 cm. In the third stage, the lymph nodes were enlarged and the inferior vena cava was thrombolytic. The abundance of blood vessels in tumor body can be reflected in CT image to a certain extent. The application of intelligent denoising algorithm preserves the details of the image better, improves the visual effect of the image effectively, and improves the quality of CT medical image. The CT image accuracy of denoising algorithm used in this study is 97.5%, significantly higher than that of enhanced CT (92.5%). Xu et al. [26] analyzed the CT image denoising algorithm with low overlapping sparse coding, and the proposed method has advanced denoising performance in terms of visual quality and objective standards. The X-CT denoising algorithm was introduced into the CT images of renal tumor patients to reduce the noise of CT images and significantly improve the image clarity. Conventional ultrasound elastography showed that the yellow and red cover of the tissue around the mass was malignant, which would cause the existence of misdiagnosis. The intelligent algorithm-based ultrasound imaging showed a significant difference in the elastic coefficient of the infiltrating catheter, and the lesions were judged as benign and malignant according to the score. The diagnostic effect was higher than that of ordinary CT images, and the diagnostic accuracy was higher.

## 5. Conclusion

The application of the ICA denoising algorithm in medical X-CT images implied corresponding variables under specific conditions and achieved better image denoising effect, which can accurately diagnose renal parenchymal tumors. The PSNR of this algorithm was significantly higher than that of the mean filtering algorithm, and it had a significant effect on the edge contour of detail features. This study has significant implications for this field. However, further research is needed to develop and innovate unified medical image denoising algorithms. There are many kinds of intelligent algorithms. Different denoising algorithms show different effects in image denoising. To explore more new intelligent algorithms suitable for medical imaging, it remains to be further studied by researchers.

## Figures and Tables

**Table 1:** Subjective evaluation image criteria.

Level	Standard	Evaluation
1	Changes in image quality affect the observation	Poor
2	The deterioration of image quality can be directly observed	General
3	Slight changes in image quality were observed but did not affect the observation	Good
4	The quality of the image did not change	Excellent

**Table 2:** Comparison of clinical data of patients.

Group	Cases	Male	Female	Age (years)	Cystic renal cancer	Clear cell carcinoma of the kidney
Observation group	40	27	13	56.3 ± 5.6	28 cases	12 cases
Control group	40	21	19	57.1 ± 6.0	0	0
*T*		−0.133		0.284	—	—
*P*		0.672		0.759	—	—

**Table 3:** Comparison of diagnostic results.

Treatment	Cases	Malignant tumor	Sensitivity (%)	Specificity (%)	Accuracy
Enhanced CT	40	37	90.0	97.5	93.8%
Denoising algorithm treated-CT	40	39	95.0	97.5	96.3%^*∗*^

Note: ^*∗*^a significant difference, *P* < 0.05.

## Data Availability

The data used to support the findings of this study are available from the corresponding author upon request.
